# Evolution of a refractory prolactin-secreting pituitary adenoma into a pituitary carcinoma: report of a challenging case and literature review

**DOI:** 10.1186/s12902-021-00874-8

**Published:** 2021-10-29

**Authors:** Congxin Dai, Bowen Sun, Shusen Guan, Wei Wang, Honggang Liu, Yong Li, Jialiang Zhang, Jun Kang

**Affiliations:** 1 Department of Neurosurgery, Beijing Tongren Hospital, Capital Medical University, Beijing, 100730 China; 2 Department of Neurosurgery, Beijing Tiantan Hospital, Capital Medical University, Beijing, 100730 China; 3 Department of Pathology, Beijing Tongren Hospital, Capital Medical University, Beijing, 100730 China

**Keywords:** Prolactin-secreting pituitary adenomas, Pituitary carcinomas, Invasive, Refractory, Recurrence, Intraspinal metastasis

## Abstract

**Background:**

Pituitary carcinomas (PCs), defined as distant metastases of pituitary neoplasms, are very rare malignancies. Because the clinical presentation of PCs is variable, early diagnosis and management remain challenging. PCs are always refractory to comprehensive treatments, and patients with PCs have extremely poor prognoses.

**Case presentation:**

We describe one case of a prolactin-secreting pituitary adenoma (PA) refractory to conventional therapy that evolved into a PC with intraspinal metastasis. A 34-year-old female was diagnosed with an invasive prolactin-secreting PA in 2009 and was unresponsive to medical treatment with bromocriptine. The tumor was gross totally removed via transsphenoidal surgery (TSS). However, the patient experienced multiple tumor recurrences or regrowth despite comprehensive treatments, including medical therapy, two gamma knife radiosurgeries (GKSs), and four frontal craniotomies. In 2016, she was found to have an intradural extramedullary mass at the level of the fourth lumbar vertebra. The intraspinal lesion was completely resected and was confirmed as a metastatic PC based on histomorphology and immunohistochemical staining. The literature on the diagnosis, molecular pathogenesis, treatment, and prognosis of patients with prolactin-secreting PCs was reviewed.

**Conclusion:**

PCs are very rare neoplasms with variable clinical features and poor prognosis. Most PCs usually arise from aggressive PAs refractory to conventional therapy. There is no reliable marker to identify aggressive PAs with a risk for progression to PCs; thus, it is difficult to diagnose these PCs early until the presence of metastatic lesions. It is still very challenging to manage patients with PCs due to a lack of standardized protocols for diagnosis and treatment. Establishing molecular biomarkers and the pathobiology of PCs could help in the early identification of aggressive PAs most likely to evolve into PCs.

**Supplementary Information:**

The online version contains supplementary material available at 10.1186/s12902-021-00874-8.

## Background

Pituitary carcinomas (PCs) are defined as tumors of adenohypophyseal origin that show metastatic spread by either craniospinal dissemination or systemic metastases [1]. PCs are extremely rare and account for only 0.1 to 0.2% of all pituitary tumors [2]. PCs are usually endocrine active tumors that present with very aggressive clinical features and rapid progression. Because PCs are often refractory to conventional therapy, they represent a particular challenge to clinical practice. Due to their rarity, the clinical behavior and biology of PCs are not yet well understood. To date, fewer than 50 cases of prolactin-secreting PCs have been reported [3, 4]. Therefore, it is very useful for improving physicians’ understanding of PCs by sharing our experiences with a rare case. Herein, we present one patient with a prolactin-secreting pituitary adenoma (PA) refractory to comprehensive treatments, including multiple surgical resections, radiotherapy, and medical therapy, that evolved into a fatal carcinoma with intraspinal metastasis.

## Case presentation

**Fig. 1:**
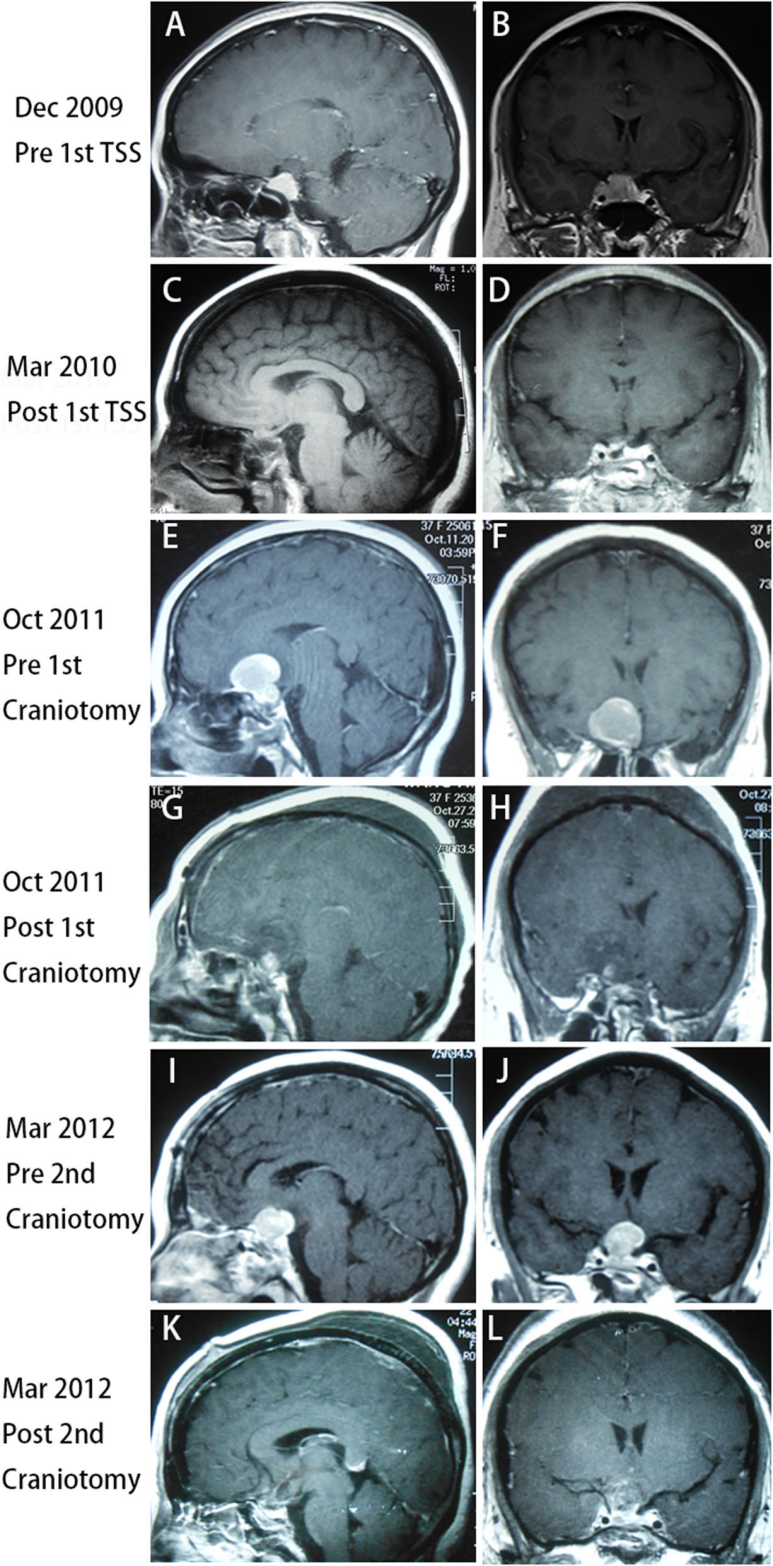
Preoperative sagittal (A) and coronal (B) planes of magnetic resonance imaging (MRI) revealed a pituitary macroadenoma 2.5 cm*2.0 cm that invaded the right cavernous sinus and encased the right internal carotid artery (ICA) completely (Knosp grade 4). (C) and (D) Three months after the first transsphenoidal surgery (TSS), MRI indicated that the tumor was gross totally resected. (E) and (F) Before the first frontal craniotomy, MRI in October 2011 revealed a rapidly enlarged tumor with compression of the optic chiasm. (G) and (H) After the first frontal craniotomy, MRI demonstrated that the tumor was subtotally removed. (I) and (J) Before the second craniotomy, MRI in March 2012 indicated a rapidly growing tumor with compression of the optic chiasm and invasion into the third ventricle. (K) and (L) After the second frontal craniotomy, the tumor was subtotal resected

A 34-year-old woman presented with headache, amenorrhea, and galactorrhea and was admitted to another hospital in December 2009. The original serum prolactin level was 700.0 ng/mL (Supplemental Fig. 1), and MRI showed a 2.5 × 2.0 cm pituitary macroadenoma, which invaded the right cavernous sinus and encased the right internal carotid artery (ICA) completely (Knosp grade 4) (Fig. 1A and B). Medical therapy using bromocriptine was immediately initiated; however, the prolactin levels were not significantly decreased (Supplemental Fig. 1). Then, transsphenoidal surgery (TSS) was performed, and gross total resection was achieved (Fig. 1C and D). Prolactinoma with a Ki-67 labeling index (LI) of 3% was diagnosed according to the pathological report, but pathological slices could not be obtained from other hospitals. After surgery, the symptoms, including headache, amenorrhea, and galactorrhea, improved significantly, and the prolactin levels decreased to the normal range (Supplemental Fig. 1). Postoperative pituitary hormone levels were basically normal. During postoperative follow-up, she experienced acute right vision loss, and MRI reported a rapidly enlarged tumor with compression of the optic chiasm (Fig. 1E and F), and the serum prolactin levels increased to 356.0 ng/mL again (Supplemental Fig. 1). She underwent first frontal craniotomy in October 2011. The tumor was subtotally removed (Fig. 1G and H), the symptoms of vision loss improved significantly after surgery, and the serum prolactin levels decreased to 356.0 ng/mL (Supplemental Fig. 1). Postoperatively, the patient was administered 0.1 mg/day desmopressin acetate for the treatment of diabetes insipidus, and the function of the anterior pituitary gland was basically normal. Unfortunately, she experienced vision loss again, and MRI in March 2012 indicated a rapidly growing tumor with compression of the optic chiasm and invasion into the third ventricle (Fig. 1I and J), and the serum prolactin levels increased to 200.0 ng/mL (samples not diluted) again (Supplemental Fig. 1). She underwent a second frontal craniotomy, and subtotal resection of the pituitary tumor was achieved (Fig. 1K and L). After surgery, the symptoms of vision loss improved significantly, and the serum prolactin levels decreased to 37.0 ng/mL again (Supplemental Fig. 1). However, laboratory findings revealed decreased serum cortisol levels of 0.08 μg/dl (reference: 5.7–22.6 μg/dl), GH levels of 0.01 μg/L (reference: 1–4, 6 μg/L), total T4 levels of 30.21 nmol/L (reference: 75–150 nmol/L) and free T4 levels of 4.07 pmol/L (reference: 7.5–15 pmol/L). The patient was administered 30 mg prednisone acetate/day and 100 μg levothyroxine sodium/day, and the hormone levels of the anterior pituitary basically returned to normal. Since then, she has received permanent hormone replacement therapy.

**Fig. 2:**
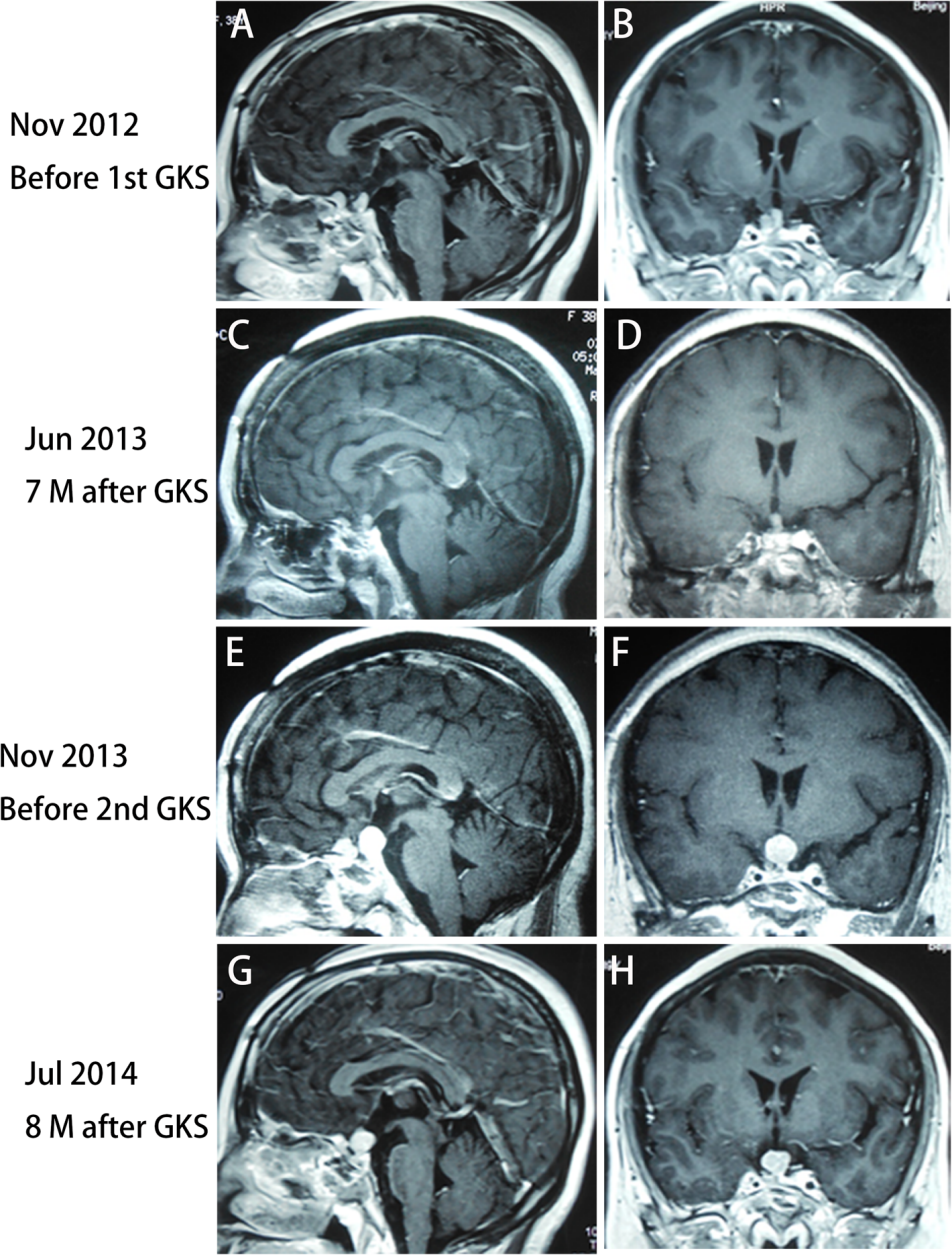
(A) and (B) Before the first gamma knife radiosurgery (GKS) treatment, MRI in November 2012 reported that the tumor was located in the sellar and suprasellar regions. (C) and (D) Seven months after the first GKS treatment, MRI revealed that the tumor size was slightly decreased. (E) and (F) Before the second GKS treatment, MRI in November 2013 demonstrated that the tumor size increased significantly again (Knosp grade 4). (G) and (H) Eight months after the second GKS treatment, MRI in July 2014 indicated that the tumor size was reduced slightly

**Fig. 3:**
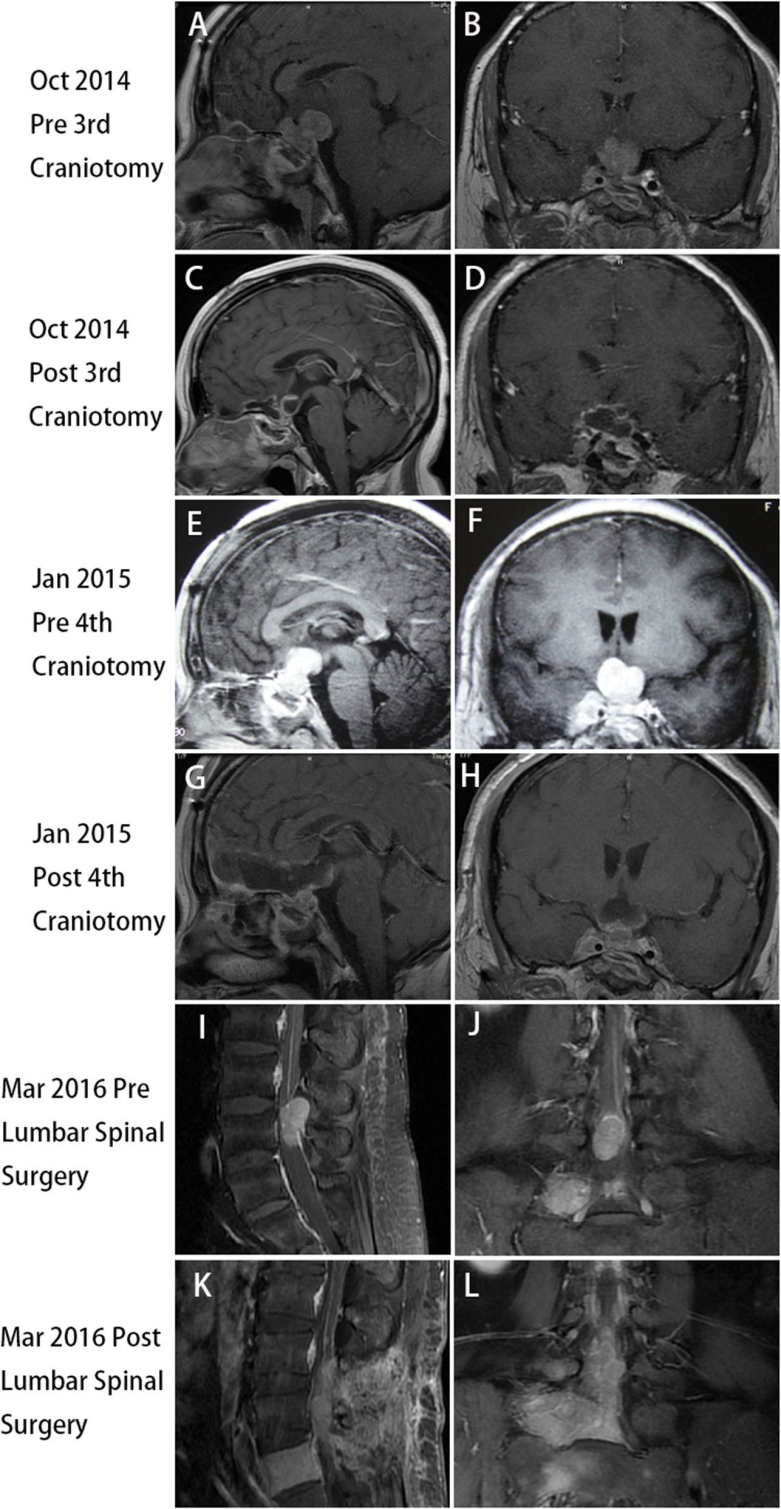
(A) and (B) Before the third frontal craniotomy, the MRI in October 2014 demonstrated a rapid enlargement of the tumor with suprasellar extension and encasing the right ICA. (C) and (D) After the third craniotomy, the tumor was subtotally removed. (E) and (F) Before the fourth frontal craniotomy, MRI in January 2015 reported that the tumor volume increased significantly and compressed the optic chiasm. (G) and (H) After the fourth craniotomy, the tumor was subtotally resected. (I) and (J) MRI of the lumbar spine indicated an intradural extramedullary mass at the level of the fourth lumbar vertebra. (K) and (L) Postoperative MRI indicated that the intradural extramedullary lesions were completely resected

**Fig. 4:**
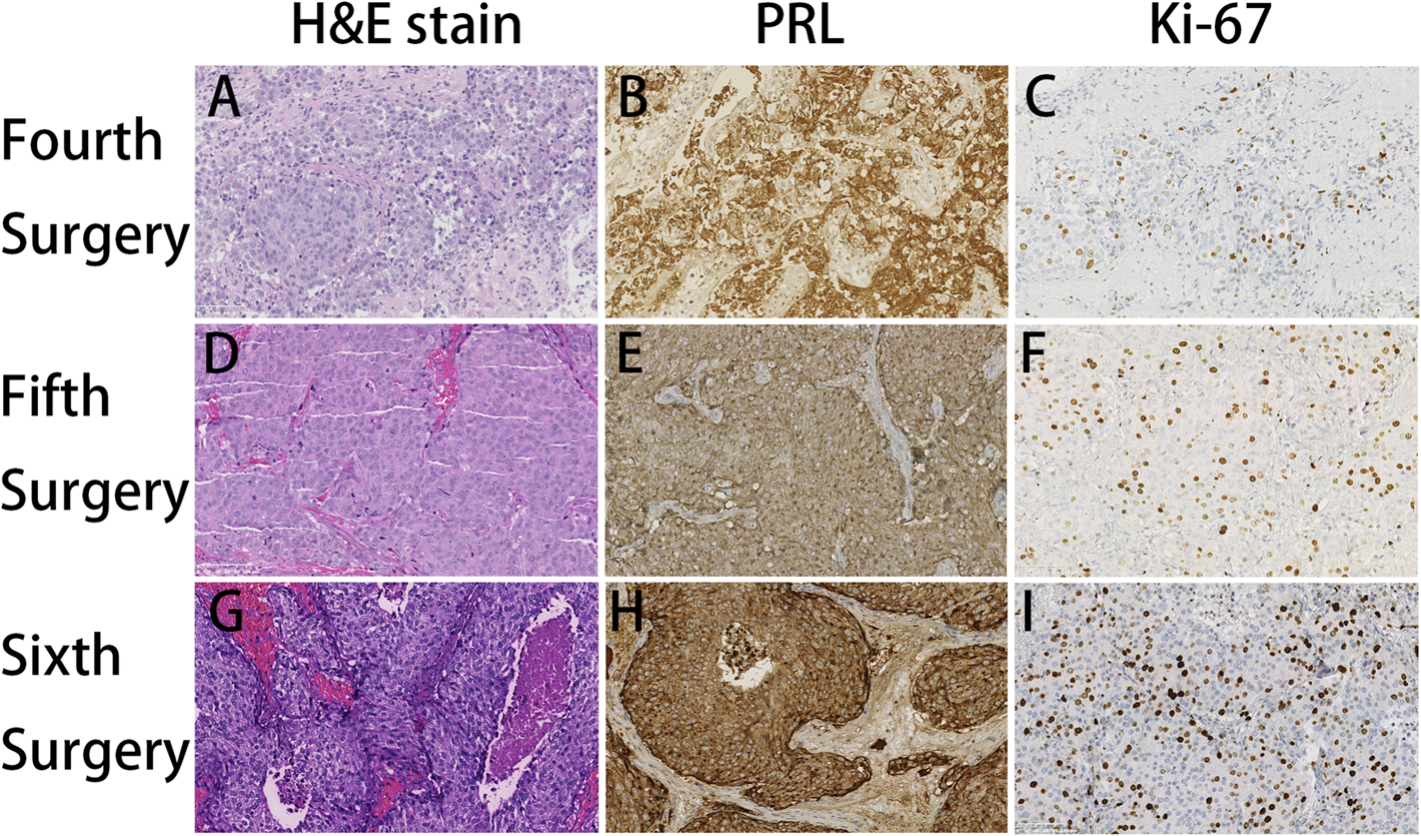
Pathological results of the resected tumor samples. (A-C) Pathological findings from the third frontal craniotomy. (A) Mitotic activity was increased (HE: 20×). (B) Immunohistochemistry (IHC) of PRL in neoplastic cells was strongly positive (20×). (C) The Ki-67 index was increased to 10% (20×). (D-F) Pathological findings from the fourth frontal craniotomy. (D) Mitotic activity was increased (HE: 20×). (E) IHC of PRL in neoplastic cells was strongly positive (20×). (F) The Ki-67 index was increased to 20% (20×). (G-I) Pathological findings from resected intraspinal tumors. (G) Mitotic activity was increased (HE: 20×); (H) IHC of PRL in neoplastic cells was strongly positive (20×); (I) The Ki-67 index was increased to 30% (20×). H&E: hematoxylin-eosin; PRL: Prolactin

Because the tumor continued to regrow or recur, she received the first gamma knife radiosurgery (GKS) treatment in November 2012 (Fig. 2A and B). Seven months after GKS treatment, MRI revealed that the tumor size was slightly decreased (Fig. 2C and D), and the serum prolactin level was reduced from 200.0 ng/mL (samples not diluted) to 57 ng/mL. However, MRI in November 2013 demonstrated that the tumor size increased significantly (Fig. 2E and F), the serum prolactin level increased to 289.0 ng/mL again (Supplemental Fig. 1), and she received the second GKS treatment. Eight months after the second GKS treatment, MRI in July 2014 indicated that the tumor size was reduced slightly (Fig. 2G and H), and the serum prolactin levels decreased to 126.0 ng/mL (Supplemental Fig. 1). Despite two GKS treatments, she had symptoms of visual field defects and vision loss in the right eye again in October 2014. The MRI demonstrated a rapid enlargement of the tumor with suprasellar extension and encasing the right ICA (Fig. 3A and B), and the serum prolactin increased to 339.0 ng/mL again (Supplemental Fig. 1). The third frontal craniotomy and subtotal removal of the tumor were performed (Fig. 3C and D), the symptoms of vision loss were improved significantly, and the serum prolactin levels decreased to 108.0 ng/mL (Supplemental Fig. 1). Pathological tests of resected tumors indicated that mitotic activity was increased, and the Ki-67 index increased to 10% (Fig. 4B and C). In November 2014, cabergoline was recommended; however, the tumor continued to enlarge, and hormones continued to increase after 1 month of treatment with cabergoline (Supplemental Fig. 1). Then, we recommended temozolomide (TMZ), but she refused this chemotherapy because the cost of TMZ was too expensive and the medical insurance con not cover its cost for patients with pituitary tumors. She experienced a loss of vision in the left eye, and MRI reported that the tumor volume increased significantly and compressed the optic chiasm (Fig. 3E and F), and the serum prolactin levels increased to 200.0 ng/mL (samples not diluted) (Supplemental Fig. 1). The fourth transcranial pituitary tumor subtotal resection was performed (Fig. 3G and H); however, vision loss did not improve, and there was a central system infection after surgery. After antibiotic treatment and lumbar drainage, the central system infection was cured. The pathological findings from resected pituitary tumors revealed that the Ki-67 index increased to 20% (Fig. 4F).

In March 2016, she presented with pain and weakness in the right leg, and MRI indicated an intradural extramedullary mass at the level of the fourth lumbar vertebra (Fig. 3I and J). Whole-body ECT reported that multiple hypermetabolic foci were found in the fifth lumbar vertebrae (Supplemental Fig. 2), and the serum prolactin levels increased to 200.0 ng/mL (samples not diluted) again (Supplemental Fig. 1). The intradural extramedullary lesions were completely resected through the posterior approach (Fig. 3K and L). After surgery, the pain and weakness in the right leg had obviously recovered, but the serum prolactin levels did not decrease significantly (Supplemental Fig. 1). Histological examination of resected intraspinal tumors showed that ICH of PRL was strongly positive but negative for other pituitary hormones, and the Ki-67 index increased to 30% (Fig. 4H and I). The pathological findings were consistent with metastatic prolactin-secreting PCs. During follow-up, the serum prolactin levels continued to increase, and the last serum prolactin test result was 356.0 ng/mL in October 2016 (Supplemental Fig. 1). Unfortunately, the tumor in the sellar area continued to progress rapidly, and the patient died in October 2017.

## Discussion and conclusion

### Classification and diagnosis of pituitary tumors

PAs account for 10–15% of all intracranial neoplasms and are the second most common primary brain tumor [5]. The prevalence of PAs is approximately 78–94 per 100,000 according to recent cross-sectional community-based studies [6, 7]. Most PAs are benign or typical adenomas, remain within the sella, are noninvasive and/or exhibit slow expansive growth, displacing surrounding tissues. PCs are defined by the presence of cerebrospinal or systemic metastases and are very rare, with an incidence of 0.1–0.2% of symptomatic pituitary tumors [8]. Although the classification system of pituitary tumors has been improved and revised rapidly in 2017, the definition of PCs has not been changed [9]. Only the presence of metastasis and the diagnosis of PCs can be determined. Therefore, it is very difficult to distinguish aggressive PAs that will develop into PCs and is especially problematic for the early diagnosis of PCs. Numerous studies have attempted to distinguish PCs from PAs early via genetic, molecular, and ultrastructural perspectives. Previous studies indicated that the Ki-67 labeling index is generally higher in PCs, which may be considered a possible molecular marker to identify tumors with potential for metastasis [10, 11]. However, other studies have not confirmed a distinction between Ki-67 in PCs and the other types of benign/typical, invasive, and aggressive PAs [12]. In this case, the Ki-67 labeling index was approximately 3% in the first surgical specimen, and it increased gradually as the operation frequency increased. Finally, it increased to 30% in metastasis at the last surgery. Additionally, PCs are intrinsically indistinguishable from PAs via pathology, immunohistochemistry, genetic analysis, and ultrastructural imaging, but these elements should be used only to supplement the diagnosis of PCs. In summary, current markers have some limitations in predicting which PAs may progress to PCs, and it is necessary to identify more reliable markers for early identification of PCs from PAs.

### Clinical presentation of PCs

PCs are very complex and heterogeneous because they present various clinical behaviors and can metastasize to different organs. PCs often arise from invasive/aggressive PAs following a protracted and complex treatment course and are diagnosed only after a metastatic focus is discovered. These PAs are often refractory to conventional treatment, including multiple operations, medical therapy, and/or radiation treatment, and undergo multiple recurrences. Patients with PCs present with symptoms similar to those in respective PAs in the early stage. The clinical presentation of PCs includes local mass effect-induced visual disturbances and headache, systemic symptoms caused by hormonal oversecretion, and/or hypopituitarism [13]. The patient in our report was initially diagnosed with an invasive prolactin-secreting PA. PAs are refractory to nearly all conventional therapies and exhibit very aggressive clinical behavior. Despite multiple surgeries, medical therapy, and radiotherapy,the present case recurred multiple times. Finally, intradural extramedullary metastasis was pathologically confirmed, resulting in a diagnosis of a PC.

In the present case, the evolution of a refractory prolactin-secreting PA into a carcinoma is consistent with previous studies [14]. The latency of invasive/aggressive PA progression to PCs can range from months to years. One study with a large series reported that the mean latency period of PA progression to PCs was 6.6 years (range: 0.3 to 18.0 years) [15]. In this case, the latency period of prolactin-secreting PA progression to carcinoma was 6.3 years, which is consistent with a previous study. However, the numbers of cases included in the previous studies are too small to confirm these conclusions, and more large-scale studies on PCs are needed.

Based on the sites and size of metastasis, PCs may present with additional site-specific symptoms or be clinically asymptomatic. The most common sites of metastasis include the central nervous system via the subarachnoid space, including the cortex, cerebellum, cerebellopontine angle and spinal subdural space, followed by extracranial sites, including the lung, liver, lymph nodes and bones. In the present case, intraspinal metastasis at the level of L-4 was confirmed pathologically and had obvious site-specific symptoms, including pain and weakness in the right leg. However, it is unknown whether vertebral lesions metastasize from pituitary tumors due to a lack of pathological support.

### Molecular pathogenesis of PCs

Although research on the molecular pathogenesis of PCs has progressed rapidly, the understanding of the molecular drivers of PA transformation into PCs is limited.

The P53 gene has been shown to have remarkably increased immunohistochemical staining in PCs compared with invasive and noninvasive PAs [16]. In PCs, p53 staining has also been found to be highly expressed in metastatic lesions compared with primary pituitary tumors [17]. Therefore, P53 may play a role in the pathogenesis of PCs. However, the function of the P53 genes in the evolution of PCs remains to be elucidated due to a lack of more clinical and basic research evidence.

Low expression of P27, as a cyclin-dependent kinase inhibitor, has been found in metastatic tumors compared with primary/recurrent lesions. One study reported a lower level of p27 protein expression in corticotroph adenomas than in normal pituitary adenomas, whereas metastatic pituitary carcinomas led to complete loss of p27 immunoreactivity [18]. Thus, loss of the tumor suppressor gene P27 may be another cause of PA transformation into PCs. However, data on the role of p27 in prolactin-producing PCs are still limited and not yet conclusive.

Although ras mutations are uncommon in pituitary tumors, they have been shown to be associated with human pituitary tumors [19]. H-ras point mutations have been described in distant metastatic PCs, indicating that H-ras gene mutations may be important in the formation of PCs and pituitary tumor metastases [20]. However, previous studies indicate that Ras mutations are rare in prolactin-secreting PAs and PCs [21], and the role of Ras mutations in the formation of prolactin-secreting PCs is still controversial.

Telomerase, as a multisubunit ribonucleoprotein responsible for cellular immortality, may play a role in the transformation of many human cancers [22]. One study reported that tumor cells acquired immortality during the course of prolactin-producing PA transformation to carcinoma, which was proven by an increase in telomerase activity and hTERT expression [23]. However, other studies demonstrated that telomere content and the expression of telomerase components are comparable between pituitary tumors and normal pituitary glands, indicating that telomere biology does not play an important role in the development of pituitary tumors [24]. Thus, more studies are needed to investigate whether telomerase participates in the transformation of PAs to PCs.

Loss of dopamine D-2 receptors has also been found in PRL-producing PCs. One study reported that intact D2R mRNA was found in primitive tumor and metastatic tissues, whereas protein for the same receptor was present only in primitive tumor tissues and not in metastatic pituitary lesions, indicating that the absence of D2R protein may play a role in an advanced stage of malignant prolactinoma.

In summary, although the pathogenesis of prolactin-producing PCs has received more attention and an increasing number of studies have been reported, the pathogenesis mechanism of PCs is still not entirely clear. Deeper research may be valuable for understanding the molecular drivers of pituitary tumor metastasis.

### Management of prolactin-secreting PCs

Because of the rarity of prolactin-secreting PCs, there have not yet been well-defined clinical guidelines for the management of prolactin-secreting PCs. The principles applied to treat aggressive/refractory prolactin-secreting PCs are also applied to carcinomas. The treatment modalities for prolactin-secreting PCs include surgical resection for the primary pituitary mass and symptomatic metastases, dopamine agonists, radiotherapy, and chemotherapy.

### Surgical treatment

Surgical resection is the first-line treatment for prolactin-secreting PCs refractory to dopamine agonists. Although gross total resection is usually not achieved due to the local invasion of the large tumor into surrounding structures and multiple distant metastases, surgical debulking can relieve compressive symptoms and decrease excess hormone secretion. PCs are usually locally invasive into the surrounding structures and therefore compress the optic chiasm, pituitary gland, pituitary stalk, cranial nerves, and major cerebral blood vessels. The most important value of surgery in the sellar region is to decompress the mass effects and relieve compressive symptoms. Intracranial metastatic lesions located within the third and/or fourth ventricles always lead to hydrocephalus, and resection of these lesions is very useful to relieve hydrocephalus symptoms. Additionally, surgical resection of intradural extramedullary metastatic lesions is critical to relieve compression of metastases on the spinal cord or nerve roots. Surgical debulking of tumors could also reduce excess prolactin hormone secretion and relieve systemic symptoms caused by hyperprolactinemia. Furthermore, surgical debulking of tumors can also enhance the other therapeutic efforts of systemic and/or radiotherapy to achieve tumor control. Therefore, although surgery is very rarely curative and there are no prospective controlled clinical reports of the long-term effects of surgery, it is still very valuable to immediately relieve the symptoms of tumor compression.

### Dopamine agonists

For patients with prolactin-secreting tumors, dopamine agonists are the primary therapy, and up to 80 to 90% of prolactinomas can be controlled in terms of tumor reduction and hormonal normalization [25]. However, prolactin-secreting PCs are usually refractory to treatment with dopamine agonists from the beginning and typically “escape” dopamine suppression during therapy [26]. Furthermore, higher doses of dopamine agonists are needed to achieve similar or weaker effects in prolactin-secreting PCs than in prolactinomas [27]. Therefore, although partial biochemical and tumor responses to treatment are observed with dopamine agonists, they offer only palliation in the treatment of prolactin-secreting PCs.

### Radiation therapy

If surgical resection and/or medical therapy fails to control prolactin-secreting PCs, radiation therapy (RT) is explored to prevent regrowth of subtotally resected tumors, including sellar tumors and distant metastatic carcinomas. It can also be used for patients with prolactin-secreting PCs who cannot undergo a surgical procedure or in whom surgical excision is not possible. For patients with prolactin-secreting PCs, radiotherapy has been shown to prevent additional tumor growth and metastasis and even result in partial remission or complete remission. The various forms of radiation therapy include conventional external beam radiotherapy and stereotactic radiosurgery, such as gamma knife radiosurgery, CyberKnife and proton beam and proton beam therapy. Because PCs are very rare, there are currently no controlled studies comparing the efficacy of conventional external beam radiotherapy and stereotactic radiosurgery for prolactin-secreting PCs. More large series clinical studies are needed to further compare the effects of different forms of radiotherapy for prolactin-secreting PCs.

### Chemotherapy

Although there are several single-agent and combination chemotherapy regimens that have been tried for prolactin-secreting PCs, most cytotoxic agents have yielded poor long-term benefits. Because there have not been any randomized studies, no consensus on a standardized protocol of chemotherapy has been published for prolactin-secreting PCs. Various cytotoxic agents and combinations include temozolomide, cisplatin, carboplatin, etoposide, procarbazine, dacarbazine, paclitaxel, vincristine, methotrexate, cyclophosphamide, doxorubicin, cyclo-hexyl-chloroethyl-nitrosourea (CCNU) in combination with 5-fluorouracil (5FU), cisplatin/carboplatin plus etoposide, and TMZ in combination with capecitabine [11]. Among these agents, TMZ has been the most widely reported and has shown acceptable response rates for patients with prolactin-secreting PCs. A recent meta-analysis reported that the objective response rate was 65.2% for patients with PC, with a median duration of response of 30 months (range, 5.5–120 months) [28]. Another systematic review demonstrated that a partial response in tumor size was evident in 66.67% of prolactin-secreting carcinomas, and a significant decrease in hormone hypersecretion was observed in 75% of patients [29]. Although the early response rates to TMZ are promising, most prolactin-secreting carcinomas fail to respond to TMZ and even acquire TMZ resistance during TMZ treatment [30]. Although TMZ has been recommended as a first-line chemotherapy in the management of PCs by the European Society of Endocrinology [31], its long-term effect is less favorable.

In summary, surgery is necessary for achieving local control and decompressing vital structures for refractory PAs with compressive symptoms. Surgery is beneficial for patients with rapidly growing tumors by decompressing the mass effect and/or reducing the tumor burden to improve the effectiveness of adjuvant therapies, but most refractory PAs usually regrow or recur after surgery. Therefore, other therapeutic approaches are always needed. If surgery fails to control tumor growth, RT is currently the next treatment option. Both conventional external beam radiotherapy and SRS provide excellent tumor control for residual or recurrent PAs, but hypopituitarism represents the most reported late complication of RT. Medical therapy plays an increasingly important role in the management of refractory PAs; however, refractory PAs are always resistant to medications, and a subset of them ultimately progress to PCs.

### Future potential therapeutic options

Previous studies demonstrated that vascular endothelial growth factor (VEGF) is associated with the development, invasion, and recurrence of pituitary tumors and is a potent therapeutic target for pituitary tumors [32]. It has been reported that one case of pituitary corticotroph carcinoma was treated with anti-VEGF (bevacizumab) and presented with long-term (26 months) disease stabilization [33]. Another study also reported that over 5 years of progression-free survival was achieved in another case of pituitary corticotroph carcinoma after treatment with concurrent chemoradiation therapy that combined TMZ and bevacizumab [34]. To date, it has been reported that bevacizumab as monotherapy, or in combination with TMZ, with TMZ and radiotherapy, with pasireotide, might be a promising alternative therapy for PCs refractory to conventional treatments [35]. However, the efficacy of anti-VEGF therapy in PCs still needs further verification due to the lack of large-scale clinical trials.

The PI3K/AKT/mTOR signaling pathways have also been previously reported to play an important role in tumor formation and progression of pituitary tumors [36]. It has been reported that mTOR inhibitor (everolimus) monotherapy achieved clinical improvement and stability for more than 6 months in one patient with PC refractory to multiple surgeries, radiation, and chemotherapy [37].

As a promising therapeutic approach, immunotherapy has been experimentally applied for many tumors, including pituitary tumors. It has been reported that a significant reduction in hormone levels and shrinkage of tumor size of primary and metastatic lesions were observed in one pituitary corticotroph carcinoma after treatment with ipilimumab and nivolumab [38]. However, another study reported that one case of pituitary ACTH-secreting adenoma failed to respond to anti-PD-1 treatment and progressed rapidly after four cycles of pembrolizumab [39]. Therefore, the role of immunotherapy in the treatment of PCs is still controversial and needs more preclinical studies and a lack of large-scale clinical trials for further evaluation.

In summary, prolactin-secreting PCs are very rare neoplasms with poor prognosis, and their early diagnosis and treatment are still very challenging. We present a case of a female patient with invasive prolactin-secreting PAs refractory to conventional treatments, including multiple operations, medical therapy, and GKS, and finally evolved into a fatal carcinoma with intraspinal metastasis. This paper highlights the difficulty in the management of PCs and the morbidity and mortality associated with PCs. Because the clinical presentation of PCs is variable, early identification of aggressive PAs at risk for progression to PCs is difficult. Although research on the pathogenesis of prolactin-producing PCs has progressed rapidly, the understanding of their pathogenesis mechanism is still not entirely clear. Various chemotherapies have been utilized in PCs, and TMZ has shown success and has been recommended as a first-line chemotherapy for PCs. Potential therapeutic approaches, including anti-VEGF therapy, targeted therapy and immunotherapy, have shown promise in case reports, and further study is required to elucidate the pathogenesis of PCs and determine the best therapeutic strategies for PCs.

## Supplementary Information

**Additional file 1.**

**Additional file 2.**

## Data Availability

The datasets used and/or analyzed during the current study are available from the corresponding author on reasonable request.

## Abbreviations

PCs: Pituitary carcinoma
PAs: Pituitary adenomas
TSS: Transsphenoidal surgery
ICA: Internal carotid artery
GKS: Gamma knife radiosurgery
RT: Radiation therapy
CCNU: Cyclo-hexyl-chloroethyl-nitrosourea
5FU: 5-Fluorouracil
TMZ: Temozolomide
VEGF: Vascular endothelial growth factor
