# Supplementary figures and images for: Evolution of a refractory prolactin-secreting pituitary adenoma into a pituitary carcinoma: report of a challenging case and literature review
Source: BMC Endocr Disord. 2021 Oct 29;21:217. doi: 10.1186/s12902-021-00874-8 (PMC8555299; doi:10.1186/s12902-021-00874-8)

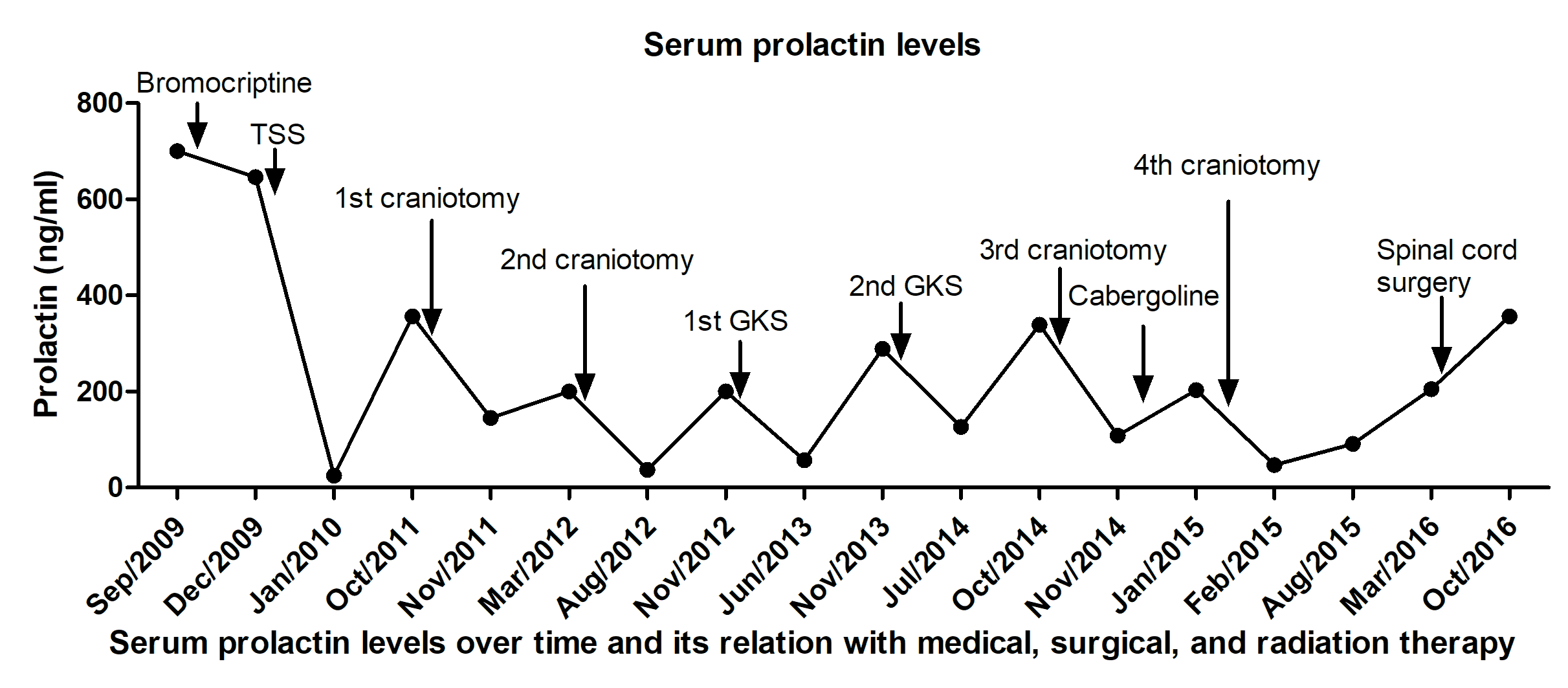

Supplement: Supplementary file 1 — Additional file 1. [file 12902_2021_874_MOESM1_ESM.tif]

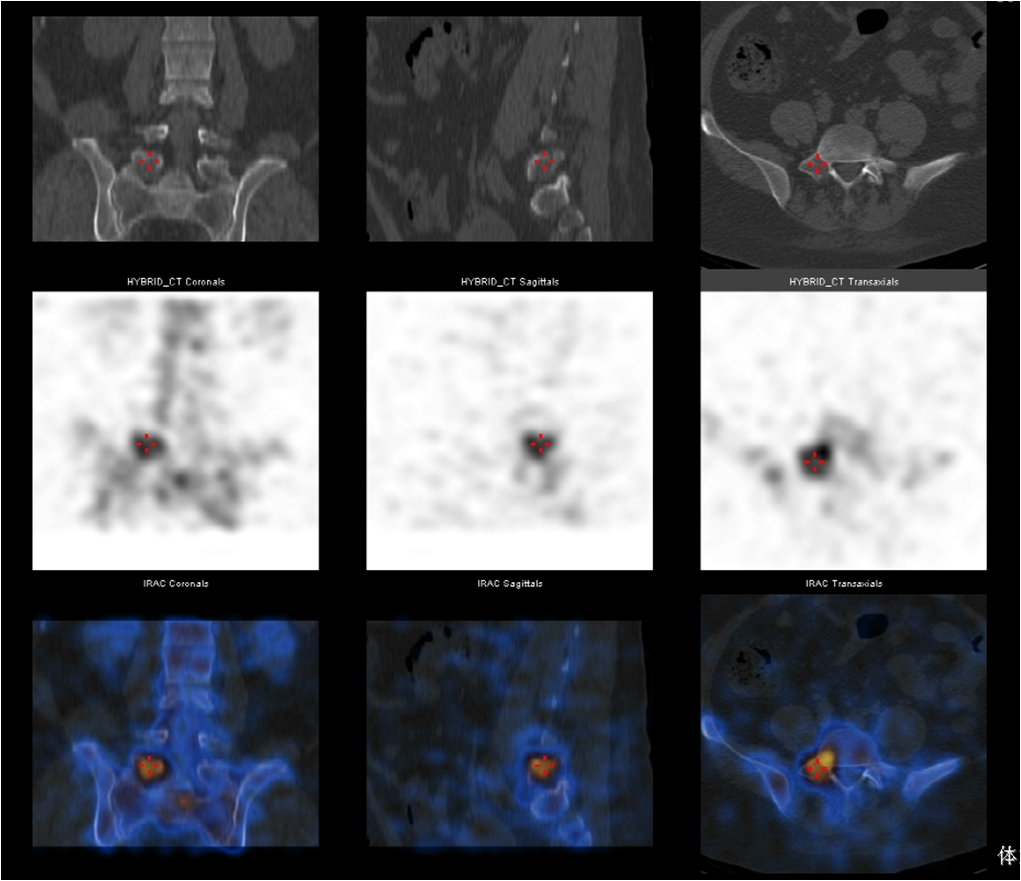

Supplement: Supplementary file 2 — Additional file 2. [file 12902_2021_874_MOESM2_ESM.tif]
